# A new method for release of severe mentosternal contractures under central neuraxial blockade

**DOI:** 10.4103/0970-0358.70723

**Published:** 2010-09

**Authors:** Vishal Mago, V. B. Singh

**Affiliations:** Plastic Surgery Unit, Govt. Medical College & Dr. S.T.M Government Hospital, Haldwani, Uttarakhand, India; 1 Department of Anesthesia, Govt. Medical College & Dr. S.T.M Government Hospital, Haldwani, Uttarakhand, India

**Keywords:** Burn contractures, neck, thoracic epidural anesthesia

## Abstract

A new method for release of severe mentosternal contractures has been described in this paper under central neuraxial blockade. The contracture release was performed under thoracic epidural analgesia. This technique can benefit patients with mentosternal contractures to avoid the problems of entubation and it can also assist in postoperative recovery and analgesia. The epidural catheter can be used to extend the height or duration of intraoperative block and is also useful to provide postoperative epidural analgesia.

## INTRODUCTION

Release of neck contractures has always been a challenge for anaesthetists because of the difficult airway position of the patient and the side effects of endotracheal intubation. Central neuraxial analgesia has become an indispensable technique to provide analgesia to surgical patients.[1] However, there are clinical situations wherein patient preference, patient physiology or the surgical procedure makes central neuraxial blockade the procedure of choice. There is also growing evidence that this technique may improve the outcome in selected situations.[2] The authors used this new and novel method of thoracic epidural neuraxial blockade for the release of severe mentosternal contractures in four children and one case of post epilepsy burn with nonhealing ulcers over back and left shoulder (skin grafting was done). This method avoids the hazards of endotracheal intubation, gives an extended period of postoperative relief of pain and epidural top up increases block height.

## MATERIALS AND METHODS

The study was done in five patients with severe mentosternal contractures, who were familiarised with and consented to the thoracic epidural technique. Routine pre-anaesthetic check-up of all the patients was done. Absolute contraindications to neuraxial blockade include parent refusal, systemic infection, local infection at the site, coagulopathy and thrombocytopenia. Anatomical anomalies were excluded before the procedure.

### Technique

Patients with severe mentosternal contracture were posted for release and skin grafting in routine operation theatre. After securing intravenous access, pre-anaesthetic medication was given. Patient was placed in left lateral decubitus position and local lignocaine spray was applied over the prepared site.

After proper positioning, sterile skin preparation and draping, the epidural catheter was secured at T7-8 interspace using an 18-gauge Tuohy needle keeping a midline approach and ensuring that the bevel of the needle remains lateral. Loss of resistance was achieved with local anaesthetic, and saline mixture was used to locate the epidural space. After reaching the space, 4 ml normal saline was injected to facilitate passage of the catheter.

Neck contracture release was done and proper haemostasis was achieved. The skin grafts were stitched on recipient areas with ethilon suture and postoperative plaster of Paris splint was applied. Postoperative recovery was uneventful. The epidural catheter was removed 72 hours after surgery.

## DISCUSSION

Administering anaesthesia to patients with severe neck contractures is probably the most hazardous procedure in reconstructive burn surgery. Laryngeal intubation ranges from difficult to impossible. Mask ventilation also may be compromised.[3] The scar contracture release may first be done under local anaesthesia and then intubation performed, but this adds to the morbidity. Our new method overcomes all the previous limitations and helps in easy execution of the operative plan with good postoperative analgesia. Central neuraxial analgesia has become an indispensable technique to provide analgesia to non surgical patients. However, there are clinical situations wherein patient preference, patient physiology or the surgical procedure makes central neuraxial blockade the procedure of choice.[2]

Thoracic epidural analgesia has become the standard method in postoperative pain control after thoracotomy. Willschke *et al*. reported the advantages of ultrasound-guided placement of epidural catheters to be faster, with a reduction in bone contact, direct visualisation of neuraxial structures and better outline of the spread of local anaesthetic into space.[4] Ryu *et al*. recommend an obtuse angle approach to maximise the chance of reach of epidural catheter at the intended level. The obtuse approach angle provides a longer coiling length.[5]

Regional anaesthesia in children provides both intraoperative and postoperative relief of pain. It is useful in clinical situations where general anesthesia is technically difficult or in children with chronic obstructive pulmonary disease (COPD) or myopathy. The use of skeletal muscle relaxants can be avoided with epidural block. The sympathetic blockade associated with epidural anaesthesia enhances surgical exposure by constricting the bowel.[2] There is avoidance of significant cardiac depression. Airway protective reflexes are intact. Postoperative pain relief can be provided without the use of narcotics. Tobias et al suggests that thoracic epidural is a safe and effective method of postoperative analgesia following thoracic surgery in children.[6]

Continuous epidural anaesthesia offers almost unlimited duration for surgery. There is no dural puncture in epidural anaesthesia so there is no postoperative spinal headache. Epidural anaesthesia is sometimes patchy as the solution travels through a tissue plane in contrast to a fluid-filled compartment.

Thoracic epidural analgesia (TEA) provides good protection from stress response, ensures haemodynamic stability, allows early extubation, improves the distribution of coronary blood flow and reduces the demand for oxygen.[7]

Thoracic epidural analgesia is a safe and effective method of postoperative analgesia for children subjected to open heart surgery. Ease of removal of the stylet, ease of injection, and negative aspiration and test doses predict successful placement and obviate the need for routine radiographic confirmation of catheter position.[8] The combination of TEA and general anaesthesia has been shown to improve pulmonary function, increase blood supply and has a beneficial effect on gut motility in both surgeries of upper and lower GI tract.

Scherer *et al*. reported no neurological sequelae of thoracic epidural catheters. Primary perforation of the dura occurred in 13 patients.[9] The incidence of complications such as transient postoperative paraesthesias was seen to be lower due to the shorter catheter threading distance. The incidence of dural puncture occurs with less than 1% frequency in experienced hands. Meticulous attention to sterile technique is vital for reducing infectious complications.

The authors report an excellent technique to overcome the side effects of difficult intubation. Flexibility in approach, frequent assessment, vigilance in monitoring, and recognition of potential complications are important to ensure the success of whichever modality is chosen.

## References

[1] Bernards CM, Barash PG, Cullen BF, Stoelting RK (2006). Epidural and spinal anaesthesia. Clinical Anaesthesia.

[2] Prithvi Raj P (2002). Textbook of Regional Anaesthesia.

[3] Cohen M (1995). Mastery of Plastic and Reconstructive Surgery.

[4] Willschke H, Marhofer P, Bösenberg A, Johnston S, Wanzel O, Sitzwohl C (2006). Epidural catheter placement in children: Comparing a novel approach using ultrasound guidance and a standard loss-of-resistance technique. Br J Anaesth.

[5] Ryu HG, Bahk JH, Lee CJ, Lim YJ (2007). The coiling length of thoracic epidural catheters: The influence of epidural approach angle. Br J Anaesth.

[6] Tobias JD, Lowe S, O’Dell N, Holcomb GW (1993). Thoracic epidural anaesthesia in infants and children. Can J Anaesth.

[7] Olivier JF, Le N, Choinière JL, Prieto I, Basile F, Hemmerling T (2005). Comparison of three different epidural solutions in off pump cardiac surgery: Pilot study. Br J Anaesth.

[8] Gunter JB, Eng C (1992). Thoracic epidural anaesthesia via caudal approach in children. Anaesthesiology.

[9] Scherer R, Schmutzler M, Giebler R, Erhard J, Stöcker L, Kox WJ (1993). Complications related to thoracic epidural analgesia:A prospective study in 1071 surgical patients. Acta Anaesthesiol Scand.

